# *N*-Methylphthalimide-substituted benzimidazolium salts and PEPPSI Pd–NHC complexes: synthesis, characterization and catalytic activity in carbon–carbon bond-forming reactions

**DOI:** 10.3762/bjoc.12.9

**Published:** 2016-01-15

**Authors:** Senem Akkoç, Yetkin Gök, İlhan Özer İlhan, Veysel Kayser

**Affiliations:** 1 Faculty of Pharmacy, The University of Sydney, 2006 Sydney, Australia; 2 Department of Chemistry, Faculty of Sciences, Erciyes University, Talas Street, 38039 Kayseri, Turkey; 3 Department of Chemistry, Faculty of Arts and Sciences, Inönü University, 44280 Malatya, Turkey

**Keywords:** arylation, benzimidazolium salts, catalysis, N-heterocyclic carbene, PEPPSI complex, Suzuki–Miyaura cross-coupling reaction

## Abstract

A series of novel benzimidazolium salts (**1–4**) and their pyridine enhanced precatalyst preparation stabilization and initiation (PEPPSI) themed palladium *N*-heterocyclic carbene complexes [PdCl_2_(NHC)(Py)] (**5–8**), where NHC = 1-(*N*-methylphthalimide)-3-alkylbenzimidazolin-2-ylidene and Py = 3-chloropyridine, were synthesized and characterized by means of ^1^H and ^13^C{^1^H} NMR, UV–vis (for **5–8**), ESI-FTICR-MS (for **2**, **4**, **6–8**) and FTIR spectroscopic methods and elemental analysis. The synthesized compounds were tested in Suzuki–Miyaura cross-coupling (for **1–8**) and arylation (for **5–8**) reactions. As catalysts, they demonstrated a highly efficient route for the formation of asymmetric biaryl compounds even though they were used in very low loading. For example, all compounds displayed good catalytic activity for the C–C bond formation of 4-*tert*-butylphenylboronic acid with 4-chlorotoluene.

## Introduction

The use of N-heterocyclic carbenes (NHCs) as ligands was started by Wanzlick [1] and Öfele [2] almost fifty years ago. There have been major advances in the design and synthesis of metal complexes containing N-heterocyclic carbene ligands in the last two decades, and they had a wide range of applications in different fields, particularly in homogeneous/heterogeneous catalysis [3–8] and bioorganometallic chemistry [9–11]. This is because NHC complexes are easily obtained by deprotonating imidazolium or benzimidazolium salts and most are relatively stable in air and moisture. They are weak π-acceptors and strong σ-donors and can form strong M–C bonds with transition metal ions compared to trivalent phosphine ligands [12–13].

As catalysts, palladium N-heterocyclic carbene (Pd–NHC) complexes display remarkable activities in coupling reactions [5,14–17]. Among various Pd–NHC complexes such as [Pd(NHC)(dmba)Cl] (dmba = *N*,*N*-dimethylbenzylamine) and [Pd(NHC)(Im)Cl_2_] (Im = imidazole) [18–19], [PEPPSI Pd–NHC] complexes are prevalent due to the combination of efficiency and versatility [20–21]. The synthesis conditions for these complexes are generally mild and do not require an inert atmosphere. The steric and electronic parameters are also easily modified by attaching substituents. PEPPSI Pd–NHC complexes have been used in different coupling reactions such as Mizoroki–Heck cross-coupling [22–23], Suzuki–Miyaura cross-coupling [24–25], Sonogashira [26] and arylation reactions [27].

There are suitable precatalyst scaffolds, which were developed by Nolan [28], Organ [21] and Buchwald [29]. To find more effective catalysts containing an Organ type scaffold among these precatalyst scaffolds, we synthesized four new pure *N*-methylphthalimide substituted benzimidazolium salts (**1–4**) and their PEPPSI Pd–NHC complexes (**5–8**) in this study. The structures of all compounds were confirmed by various spectroscopic methods (^1^H and ^13^C{^1^H} NMR, UV–vis, ESI-FTICR-MS, FTIR) and elemental analysis. The PEPPSI Pd–NHC complexes were tested for catalytic activities both in direct arylation and Suzuki–Miyaura cross-coupling reactions. The catalytic activities of benzimidazolium salts were only tested in a Suzuki–Miyaura cross-coupling reaction. The compounds were found to be very efficient in the symmetric and asymmetric C–C bond-forming reactions.

## Results and Discussion

### Synthesis of *N*-methylphthalimide substituted benzimidazolium salts

**Scheme 1:**
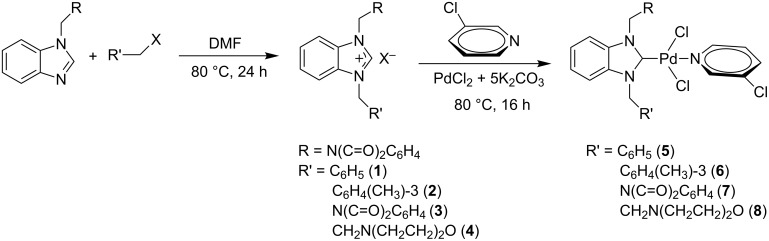
Synthesis of benzimidazolium salts and their PEPPSI Pd–NHC complexes.

New benzimidazolium salts **1–4**, which are carbene precursors, were synthesized by *N*-alkylbenzimidazole and various alkyl halides in DMF (Scheme 1). These salts, especially containing benzyl and 3-methylbenzyl groups, were obtained in very high yields (81–97%) as white or cream solids. The salt containing the 2-morpholinoethyl group was obtained in a much lower yield of 62%.

The benzimidazolium salts include an acidic NC*H*N proton, which can be deprotonated easily to form an NHC, at the C2 position of the benzimidazole ring. The sharp salt peak indicating the synthesis of a benzimidazolium salt came quite downfield at δ 10.13, 11.10, 9.65 and 10.83 ppm in the ^1^H NMR spectra for **1–4**, respectively. The N*C*HN peaks of the carbene precursors were observed at δ 144.56, 144.69, 143.55 and 145.26 ppm in the ^13^C{^1^H} NMR spectra for **1–4**, respectively. The formation of the benzimidazolium salts was also evident through their IR spectra, which showed peaks at 1562.2, 1558.4, 1562.2 and 1554.5 cm^−1^ for the ν_(CN)_ bond of **1–4**, respectively. Compounds **2** and **4** among these salts were further characterized by using high-resolution mass spectrometry (HRMS). The mass spectra demonstrated *m*/*z* peaks at 382.16 and 391.18 for the cationic moieties against the calculated value of *m*/*z* 382.43 and 391.44 for **2** and **4**, respectively.

### Synthesis of PEPPSI Pd–NHC complexes

Our aim was to synthesize novel Pd–NHC complexes containing a PEPPSI skeleton. The target PEPPSI Pd–NHC complexes **5–8** were successfully synthesized by using carbene precursors **1–4**, PdCl_2_ and K_2_CO_3_ as a base in 3-chloropyridine (Scheme 1). The colors of the obtained solid complexes were either yellow or cream. These complexes, which are stable both in solution and in solid states against air, light and moisture, were obtained in low yields of 25–60%. All complexes with a benzimidazolium moiety display characteristic signals for the 3-chloropyridine ligand in the ^1^H and ^13^C{^1^H} NMR spectra. For the ^1^H NMR spectra of the metal complexes, sharp peaks in the lower field belonging to the benzimidazolium salts (NC*H*N) were not observed between δ 10 and 12 ppm. The FTIR data clearly indicated the presence of ν_(CN)_ at 1508.2, 1446.5, 1394.4 and 1444.6 cm^−1^ for the PEPPSI Pd–NHC complexes **5–8**, respectively. The formation of a C–N module in the benzimidazole ring correlated with a shift of IR (CN) band. The complexes **6–8** were further characterized by means of HRMS which showed *m*/*z* peaks at 637.02, 691.99 and 574.99 for the cationic moieties against the calculated values of *m*/*z* 636.84, 691.84 and 574.95, respectively. Unfortunately, we were unable to yield a proper single crystal from these compounds for X-ray diffraction. Unlike the salts, the metal complexes showed absorbance in UV–vis experiments.

### Absorption spectroscopy studies

**Figure 1:**
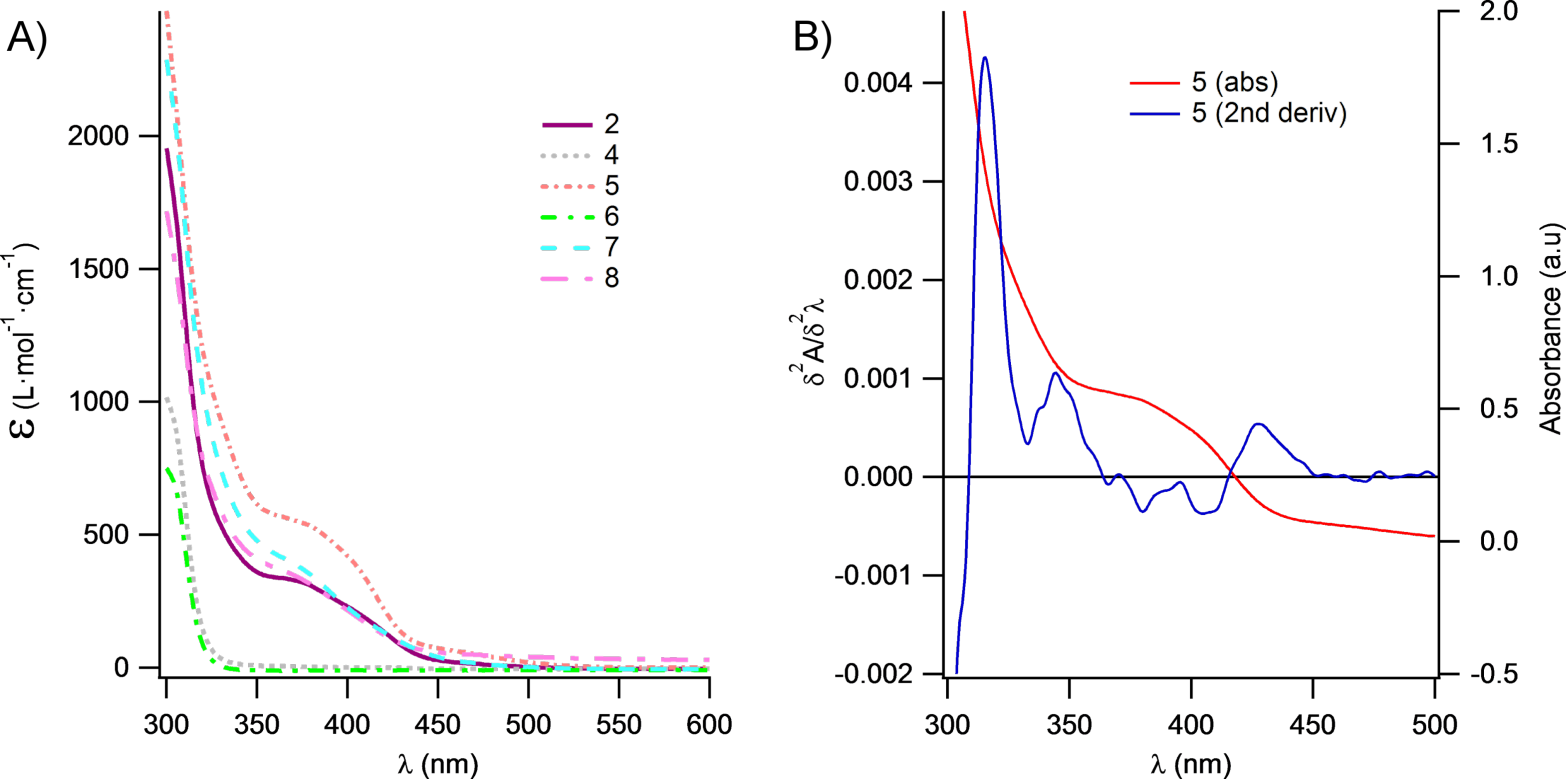
(A) UV–vis absorbance spectra were taken in DMSO. (B) The second derivative of the compound **5** calculated from A. Other metal complexes have similar second derivative bands but are omitted for simplicity.

The absorption spectra of the complexes were recorded in DMSO and are shown in Figure 1A.

Only metal complexes show an absorbance above 330 nm and have the highest absorption, whereas salts did not display any absorbance except for an intense peak below 330 nm. Therefore, we only discuss the metal complexes here. For the metal complexes, the spectra are characterized by a broad band between 350 and 430 nm and display a strong absorption below 350 nm. Complex **5** exhibits the overall highest absorption, whereas the overall absorption of complex **7** seems to be the lowest of the six investigated complexes. Very broad absorption spectra are indicative of a charge transfer, and to deconvolute the shoulder or ripple of the absorption spectra, we applied the second derivative analysis (Figure 1B). The second derivative spectra were noisy, as can clearly be seen after smoothing three positive peaks (at 315, 345 and 430 nm) and two negative peaks (at 380 and 405 nm). In the second derivative analysis, a negative band has a minimum at the same wavelength as the maximum on the main absorption spectrum. With this in mind, there are at least two absorbance bands buried between 350–430 nm.

### Catalytic activity of PEPPSI Pd–NHC complexes as catalysts in arylation reaction

First, we performed the arylation reaction between the 4-bromoacetophenone, which is electron-deficient, with 2-*n*-butylthiophene without the catalyst at 110 °C for 1 h in DMAc as solvent and the reaction resulted in only a 1% yield. When we attempted the same reaction using **8** as a catalyst at 130 °C, the efficiency of the reaction was 27% (Table 1, entry 3). For catalyst **5**, the yield dropped to 9%, the temperature was decreased to 110 °C and the procedure time was increased from 1 h to 1.5 h (Table 1, entry 2).

**Table 1:** PEPPSI Pd–NHC catalyzed direct intermolecular arylation of heteroaryl derivatives with various aryl bromides^a,b^.

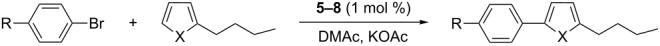						

Entry	R	X	Time (h)	Temp. (°C)	Comp.	Yield (%)

1	CH_3_(C=O)-	S	1	110	–	1
2			1.5	110	**5**	9
3			1	130	**8**	27
4		O	1	110	**5**	14
5			1	110	**6**	49
6			1	110	**7**	83
7			1	110	**8**	89
8	H-	S	1	110	**8**	97
9		O	21	80	**7**	71
10			21	90	**7**	84
11			21	110	**7**	98
12	CH_3_O-	S	1	130	**5**	79

^a^Reaction conditions: 2-*n*-butylthiophene or 2-*n*-butylfuran (2 mmol), 4-bromoacetophenone, bromobenzene or 4-bromoanisole (1 mmol), PEPPSI Pd–NHC **5–8** (1 mol %), KOAc (1 mmol), DMAc (2 mL), 80–130 °C, 1 or 21 h. Product purity was checked by GC and NMR. ^b^Yields were calculated according to aryl bromides.

When 4-bromoacetophenone was used as a substrate with catalyst **6**, sp^2^–sp^2^ C–C bond formation with 2-*n*-butylfuran was achieved with a yield of 49% in just 1 h (Table 1, entry 5). The product was obtained in a much lower yield when the same reaction was carried out with **5** as a catalyst. Compounds **7** and **8** as catalysts were displaying better results than complexes **5** and **6** for the same reaction (Table 1, entries 6 and 7). When we employed electron-neutral bromobenzene as a substrate instead of 4-bromoacetophenone, C–C bond formation (2-butyl-5-phenylthiophene) was achieved in 97% yield using catalyst **8** at 110 °C with a reaction time of 1 h (Table 1, entry 8). When bromobenzene was used with catalyst **7** at 80 °C for 21 h, the product 2-butyl-5-phenylfuran was obtained in 71% yield. By increasing the reaction temperature by 10 degree centigrade, as shown in entry 10, the result showed a reaction yield that was 13% higher than that of entry 9. However, when we further increased the temperature by more than 30 degrees to 110 °C, a maximum yield of 98% was obtained (Table 1, entry 11). In the reaction catalyzed by **5**, 2-butyl-5-(4-methoxyphenyl)thiophene was obtained in 79% yield (Table 1, entry 12).

### Catalytic activity of synthesized compounds in Suzuki–Miyaura cross-coupling reaction

The Suzuki–Miyaura cross-coupling reaction, which has mostly been performed in organic solvents until recently, can now be performed using green solvents under mild conditions [29–34]. We used H_2_O with DMF as solvents in different proportions in this work. We preferred to use a 1:1 ratio in the Suzuki–Miyaura reaction as there was not much difference between the obtained results when the ratios used were 3:1 or 1:1. To optimize the reaction conditions, a series of experiments with different bases such as KOH, NaOH and K_2_CO_3_ were conducted at different temperatures to provide the coupling of the C–C bond of different substrates with phenylboronic acid. The best results were obtained with the base KOH. We also employed different time periods ranging from 1 to 3 h. When the reaction time was extended, the yield increased in a linear manner.

The Suzuki–Miyaura reaction was carried out using electron-rich (4-chloroanisole, 4-chlorotoluene), electron-poor (4-chloroacetophenone, 4-chloronitrobenzene) and electron-neutral (chlorobenzene) substrates. We studied the catalytic activity of compound **1** regarding C–C bond coupling of 4-iodoacetophenone as a substrate with phenylboronic acid (Table 2, entry 2). This catalytic system showed a better performance for aryl iodide than for aryl chlorides except chlorobenzene (Table 2, entries 1–17). 4-Acetylbiphenyl was obtained in 99% yield and 100% conversion at 80 °C and 1 h (Table 2, entry 2). The best results were obtained when chlorobenzene was used as a substrate (Table 2, entries 18–21) whereas least favorable results were obtained when the electron-poor substrates were used. Among the employed carbene precursors, compound **4** gave the best result to acquire the 4-nitrobiphenyl product (Table 2, entries 8 and 9). Compounds **2** and **4** gave very good results to get the 4-methoxybiphenyl product from among the used carbene precursors (Table 2, entries 11 and 13).

**Table 2:** Suzuki–Miyaura cross-coupling reaction of phenylboronic acid with aryl chlorides^a,b^.

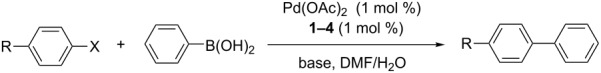									

Entry	R-C_6_H_4_-X	LHX	Base	Time (h)	Temp (°C)	DMF (mL)	H_2_O (mL)	Yield (%)	Conv. (%)

1	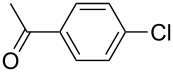	**1**	KOH	1	80	3	1	41	53

2	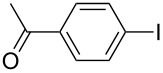	**1**	KOH	1	80	3	1	99	100

3	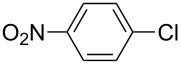	**1**	KOH	1	80	3	1	27	42
4		**2**	KOH	2	80	2	2	34	40
5		**2**	KOH	3	80	2	2	74	77
6		**3**	KOH	2	80	2	2	25	62
7		**3**	KOH	3	80	2	2	30	77
8		**4**	KOH	2	80	2	2	75	76
9		**4**	KOH	3	80	2	2	75	97

10	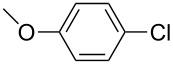	**1**	KOH	1	80	3	1	52	68
11		**2**	KOH	2	80	3	1	94	99
12		**3**	KOH	2	80	3	1	23	55
13		**4**	KOH	2	80	3	1	95	98

14	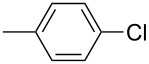	**1**	KOH	1	80	3	1	90	92
15		**2**	KOH	1	80	2	2	93	99
16		**3**	KOH	1	80	2	2	44	92
17		**4**	KOH	1	80	2	2	12	99

18	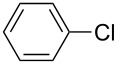	**1**	KOH	2	70	2	2	99.9	99.9
19		**1**	NaOH	2	70	2	2	99.8	99.8
20		**1**	K_2_CO_3_	2	70	2	2	96	98
21		**1**	KOH	1	80	3	1	99.9	100

^a^Reaction conditions: *p*-R-C_6_H_4_Cl (1.0 mmol), Pd(OAc)_2_ (1.0 mol %), phenylboronic acid (1.5 mmol), base (2.0 mmol), **1–4** (1 mol %), DMF/H_2_O, 70–80 °C, 1–3 h. ^b^Yields were calculated according to aryl chlorides by using GC or GC–MS.

Melvin et al. found the coupling of phenylboronic acid with 4-chlorotoluene with a yield of approximately 45% within 90 minutes at room temperature and reaction conditions of 1.0 mol % PEPPSI-IPr, K_2_CO_3_, MeOH/THF (19:1). They obtained the same coupling product with a yield of around 50% when they changed the amount of PEPPSI-IPr to 0.5 mol %, the base to KO*t*-Bu and extended the reaction time to 2 h [35]. However, we obtained the same product in much higher yields such as 90 and 93% under reaction conditions of 1 mol % of Pd(OAc)_2_, 1 mol % of **1** and **2**, KOH, DMF/H_2_O within a short period of time (1 h) at 80 °C.

The synthesized carbene precursors consist of an electron-neutral group (benzyl), electron-donating group (3-methylbenzyl) and electron-withdrawing group (*N*-methylphthalimide) on the benzimidazolium salts. These groups are important for the catalytic performance and to understand the electronic effect of the ligands. In the presence of the catalysts formed in the in situ medium, the coupling of 4-methoxy-1-chlorobenzene with 2,5-dimethoxyphenylboronic acid was performed with overall low yields (Table 3, entries 1–4). While carbene precursor **2** showed the lowest catalytic activity, **3** gave the highest catalytic activity (Table 3, entries 2 and 3). We acquired excellent catalytic activity results using 4-*tert*-butylphenylboronic acid which is a derivative of phenylboronic acid (Table 3, entries 5–8). All compounds were found to be very effective for this coupling reaction and the product yield was between 94–100%. The C–C bond formation of thianaphthene-2-boronic acid with 4-chloronitrobenzene using an in situ formed Pd–NHC complex as catalyst resulted in low yields and conversions (Table 3, entries 9–12). The usage of the morpholinoethyl substituted benzimidazolium compound **4** with Pd(OAc)_2_ resulted in higher yields of 2-(4-nitrophenyl)benzo[*b*]thiophene compared to the usage of compounds **1–3**.

**Table 3:** Suzuki–Miyaura cross-coupling reaction of boronic acid derivatives with aryl halides^a,b^.

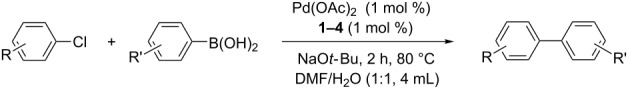						

Entry	Aryl halide	Derivatives of boronic acid	LHX	Product	Yield (%)	Conv. (%)

1	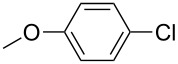	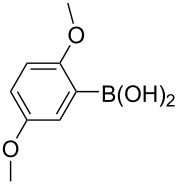	**1**	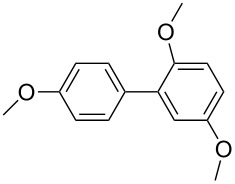	35	68
2			**2**		15	26
3			**3**		77	80
4			**4**		24	33

5	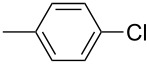	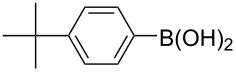	**1**	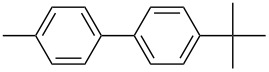	94	96
6			**2**		99	99.7
7			**3**		98	99
8			**4**		99.9	100

9	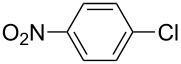	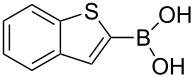	**1**	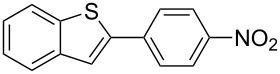	10	38
10			**2**		8	15
11			**3**		6	7
12			**4**		45	48

^a^Reaction conditions: 4-methoxy-1-chlorobenzene, 4-chlorotoluene, 4-chloronitrobenzene (1.0 mmol), Pd(OAc)_2_ (1.0 mol %), 2,5-dimethoxyphenylboronic acid, 4-*tert*-butylphenylboronic acid, thianaphthene-2-boronic acid (1.5 mmol), NaO*t*-Bu (2.0 mmol), **1–4** (1 mol %), DMF/H_2_O (1:1, 4 mL), 80 °C, 2 h. ^b^Yields were calculated according to aryl chlorides by using GC or GC–MS.

Generally, the PEPPSI Pd–NHC complexes showed similar catalytic activity with in situ formed Pd–NHC complexes under the same experiment conditions (Table 3 and Table 4). The 2-morpholinoethyl substituted Pd–NHC complex **8**, on the other hand, displayed very low activity compared to the other three complexes for the coupling of 2,5-dimethoxyphenylboronic acid with 4-methoxy-1-chlorobenzene (Table 4, entry 4). High yields were obtained in the presence of low amounts of catalysts **5–8** (1 mol %) in the coupling reaction of 4-*tert*-butylphenylboronic acid with 4-chlorotoluene (Table 4, entries 5–8). All synthesized compounds demonstrated low activity in the coupling of thianaphthene-2-boronic acid with 4-chloronitrobenzene (Table 3, entries 9–12; Table 4, entries 9–12). In C–C bond-forming reactions of different substrates with 2,5-dimethoxyphenylboronic acid and thianaphthene-2-boronic acid, complex **7** was found to be a good catalyst for the synthesis of biaryl systems in comparison to the other complexes. We observed that compounds **4** and **7** were more effective than the other compounds as catalyst (Table 3, entry 12; Table 4 entry 11).

**Table 4:** Suzuki–Miyaura cross-coupling reaction of boronic acid derivatives with aryl chlorides^a,b^.

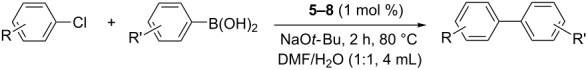						

Entry	Aryl chloride	Derivatives of boronic acid	PEPPSI Pd–NHC	Product	Yield (%)	Conv. (%)

1	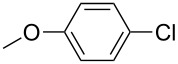	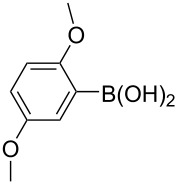	**5**	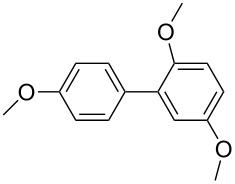	56	67
2			**6**		51	55
3			**7**		59	77
4			**8**		9	25

5	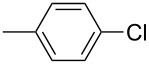	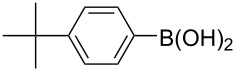	**5**	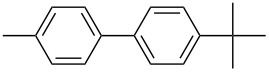	92	99
6			**6**		95	99
7			**7**		93	98
8			**8**		99.9	100

9	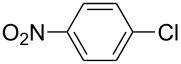	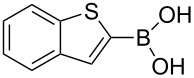	**5**	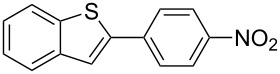	3	5
10			**6**		1	9
11			**7**		35	55
12			**8**		14	30

^a^Reaction conditions: 4-methoxy-1-chlorobenzene, 4-chlorotoluene, 4-chloronitrobenzene (1.0 mmol), 2,5-dimethoxyphenylboronic acid, 4-*tert*-butylphenylboronic acid, thianaphthene-2-boronic acid (1.5 mmol), NaO*t*-Bu (2.0 mmol), **5–8** (1 mol %), DMF/H_2_O (1:1, 4 mL), 80 °C, 2 h. ^b^Yields were calculated according to aryl chlorides by using GC or GC–MS.

## Conclusion

In the present study, a series of benzimidazolium salts (**1–4**) and PEPPSI Pd–NHC complexes (**5–8**) were successfully synthesized and their structures were confirmed via ^1^H and ^13^C{^1^H} NMR, ESI-FTICR-MS (for **2**, **4**, **6–8**), UV–vis, FTIR and elemental analysis. All the compounds exhibited good solubility in organic solvents and were tested in both arylation (for **5**–**8**) and Suzuki–Miyaura cross-coupling (for **1**–**8**) reactions. Pd-catalyzed direct intermolecular arylation was investigated using electron-poor, electron-rich or electron-neutral substrates. In general, an electron-neutral group was found to be more effective in the formation of biaryl product. Both in situ generated Pd–NHC and PEPPSI Pd–NHC complexes as catalysts were studied in Suzuki–Miyaura cross-coupling reactions without an inert atmosphere. Both complex types were quite effective in the coupling of 4-chlorotoluene with 4-*tert*-butylphenylboronic acid.

## Supporting Information

File 1 Experimental section.
