# Supplementary material for: N-Methylphthalimide-substituted benzimidazolium salts and PEPPSI Pd–NHC complexes: synthesis, characterization and catalytic activity in carbon–carbon bond-forming reactions
Source: Beilstein J Org Chem. 2016 Jan 15;12:81–8. doi: 10.3762/bjoc.12.9 (PMC4734419; doi:10.3762/bjoc.12.9)
Supplement: File 1 — Experimental section. [file Beilstein_J_Org_Chem-12-81-s001.pdf]

Supporting Information

for

N-Methylphthalimide-substituted benzimidazolium salts and PEPPSI Pd–NHC complexes: synthesis, characterization and catalytic activity in carbon–carbon bond-forming reactions

Senem Akkoç[1,2*§], Yetkin Gök[3], İlhan Özer İlhan[2] and Veysel Kayser[1*¶]

Address: [1]Faculty of Pharmacy, The University of Sydney, 2006 Sydney, Australia; [2]Department of Chemistry, Faculty of Sciences, Erciyes University, Talas Street, 38039 Kayseri, Turkey and [3]Department of Chemistry, Faculty of Arts and Sciences, Inönü University, 44280 Malatya, Turkey

Email: Senem Akkoç - senem.akkoc@sydney.edu.au; Veysel Kayser - veysel.kayser@sydney.edu.au

*Corresponding author

§Tel: +61 2 9351 2330; Tel: +90 352 437 52 62; Fax: +90 352 437 49 33

¶Tel: +61 2 9351 3391; Fax: +61 2 9351 4391

Experimental section

General considerations

N-Methylphthalimide-substituted novel benzimidazolium salts were synthesized under argon gas by using a Schlenk line technique. *N*,*N*-Dimethylformamide (DMF) was dried over P_4O_{10}. All reactions for the preparation of the PEPPSI Pd–NHC complexes and their purifications were carried out under ambient conditions. All reagents and solvents were purchased from Merck, Sigma-Aldrich or Scharlau.

All ^{1}H and ^{13}C{^{1}H} NMR were performed in $CDCl_3$ and DMSO-d_6 on Bruker 300 or 400 MHz Ultra Shield NMR spectrometer at ambient temperature. Chemical shifts (δ) are given in ppm relative to tetramethylsilane (TMS) as an internal reference. Coupling constants (*J*) are given in Hertz (Hz). ^{1}H NMR signals are labeled as singlet (s), triplet (t) and multiplet (m). Gas chromatography (GC) and gas chromatography–mass spectrometry (Shimadzu, GC–MS-QP2010 Plus) were used in the catalytic experiments. GC analysis was undertaken using an Agilent 6890N Network GC System with a HP-5 column (column diameter 0.32 mm, column length 30 m, column filler size 0.25 μm) and temperature range from −60 to 325 °C. The UV–vis spectra of the PEPPSI Pd–NHC complexes were taken in DMSO (0.01 M and 0.001 M) with a Shimadzu Pharmaspec UV-1700 spectrophotometer from 600 to 230 nm. A micro cuvette from Hellma with 1 cm path length was used for all UV–vis measurements with 2 nm slits. For high resolution mass spectrometry (HRMS), each sample (0.1 mg) was dissolved in methanol (1 mL) and injected at a rate of 150 μL/h by a Cole Palmer syringe pump into the mass spectrometer. Mass spectrometry was performed on a Bruker Apex Qe 7T Fourier Transform ion cyclotron resonance mass spectrometer equipped with an ESI/MALDI dual source in positive ion ESI mode. Melting points (mp) were measured in glass capillary tubes with an Electrothermal-9200 apparatus. Elemental analyses were performed by using a CHNS-932 LECO device. The FTIR spectra of the compounds were recorded in the 450–4000 cm^{-1} region with a Shimadzu FTIR 8400 spectrophotometer.

Synthesis of novel compounds

General preparation of benzimidazolium salts 1–4

Benzimidazole (1 mmol) and potassium hydroxide (1 mmol) were dissolved in ethyl alcohol (60 mL). The alkyl halide (1 mmol) was slowly added after the obtained reaction mixture was stirred at room temperature for 1 h. The solution was refluxed for 6 h, cooled to room

temperature and the precipitated potassium chloride was removed by filtration. The solvent was removed by distillation. The product was then crystallized, washed several times with diethyl ether and then dried in vacuo. To a solution of 1-alkylbenzimidazole (1 mmol) in dried DMF (4 mL), alkyl halide (1 mmol) was added slowly and the reaction mixture was stirred at 80 °C for 24 h under argon. After completion of the reaction, the DMF was removed by vacuum and diethyl ether (15 mL) was added to the mixture. The solid was washed with diethyl ether (2 × 15 mL) and dried under vacuum. The product was crystallized in an ethanol/diethyl ether mixture (3:1) at room temperature. The purified compounds were obtained as white or cream solids. Their structures were characterized by NMR (^{1}H and ^{13}C), FTIR, ESI-FTICR-MS (for **2** and **4**) spectroscopic methods and elemental analysis.

General preparation of PEPPSI Pd–NHC complexes 5–8

The synthesis of PEPPSI Pd–NHC complexes in a 75 mL Schlenk tube was performed by the reaction of benzimidazolium salts (1 mmol), $PdCl_2$ (1 mmol) and K_2CO_3 (5 mmol) as a base in 3-chloropyridine (3 mL) (for **5–8**). The solution was stirred at 80 °C for 16 h. After the reaction was finished, dichloromethane (10 mL) was added to the reaction mixture. The resulting solution was filtered through a pad of celite and silica gel to remove the unreacted palladium chloride and benzimidazolium salt. The solvent in the reaction medium was then removed. The resultant complexes were washed with diethyl ether (3 × 5 mL) and completely dried under vacuum. These complexes were characterized by ^{1}H and ^{13}C{^{1}H} NMR, FTIR, UV–vis, ESI-FTICR-MS (for **6–8**) spectroscopic methods and elemental analysis. The synthesized complexes have high solubility in organic solvents such as dichloromethane, dimethyl sulfoxide and chloroform. They are almost insoluble in diethyl ether and hexane.

Data for the compounds

Characterization of synthesized benzimidazolium salts and PEPPSI Pd–NHC complexes 1–8

1-(*N*-Phthalimidomethyl)-3-benzylbenzimidazolium bromide (1)

1-Benzylbenzimidazole was synthesized using benzimidazole (1 mmol), benzyl chloride (1 mmol) and potassium hydroxide (1 mmol) in ethanol (60 mL). To a solution of DMF containing 1-benzylbenzimidazole (1.2 g, 1 mmol), *N*-(bromomethyl)phthalimide (1.29 g, 1 mmol) was added and the reaction mixture was stirred at 80 °C for 24 h.

Yield: 97%, mp: 287–288 ºC, color: white. IR: 1562.2 (CN); 1716.5 and 1718.5 (C=O); 2875.7 cm^{-1} (aliphatic, C-H). ^{1}H NMR (400.13 MHz, DMSO-d_6, 298 K), δ: 5.83 (s, 2 H, $NCH_2C_6H_5$); 6.42 [s, 2 H, $NCH_2N(C{=}O)_2C_6H_4$]; 7.36-8.20 (m, 13 H, Ar-*H*); 10.13 (s, 1 H, NC*H*N). $^{13}C\{^{1}H\}$ NMR (100 MHz, DMSO-d_6, 298 K), δ: 47.79 [$NCH_2N(C{=}O)_2C_6H_4$]; 50.32 ($NCH_2C_6H_5$); 114.32, 114.54, 124.17, 124.25, 127.16, 127.51, 128.70, 128.78, 129.15, 129.36, 130.77, 130.99, 131.40, 131.74, 131.91, 134.27, 135.49 and 135.59 (Ar-*C*); 144.56 (N*C*HN); 167.27 and 167.43 [$NCH_2CH_2CH_2CH_2N(C{=}O)_2C_6H_4$]. Elemental analysis for $C_{23}H_{18}N_3O_2Br$ (448.31 g/mol) (%): Found: C: 61.76; H: 3.94; N: 9.31. Anal. Calc. C: 61.62; H: 4.05; N: 9.37.

1-(*N*-Phthalimidomethyl)-3-(3-methylbenzyl)benzimidazolium bromide (2)

1-(3-Methylbenzyl)benzimidazole was prepared, according to the same conditions and procedure as for 1-benzylbenzimidazole, from benzimidazole (1 mmol), 3-methylbenzyl chloride (1 mmol) and potassium hydroxide (1 mmol) in ethanol (60 mL). According to the same conditions and procedure as for **1**, **2** was synthesized from 1-(3-

methylbenzyl)benzimidazole (1.29 g, 1 mmol) and *N*-(bromomethyl)phthalimide (1.39 g, 1 mmol) in DMF (4 mL).

Yield: 92%, mp: 268–269 ºC, color: white. IR: 1558.4 (CN); 1716.5 and 1722.3 (C=O); 2912.3 cm^{-1} (aliphatic, C-H). ^{1}H NMR (400.13 MHz, $CDCl_3$, 298 K), δ: 2.31 [s, 3 H, $NCH_2C_6H_4(CH_3)$-3]; 5.83 [s, 2 H, $NCH_2C_6H_4(CH_3)$-3]; 6.71 [s, 2 H, $NCH_2N(C{=}O)_2C_6H_4$]; 7.11-8.05 (m, 12 H, Ar-*H*); 11.10 (s, 1 H, NC*H*N). $^{13}C\{^{1}H\}$ NMR (100 MHz, $CDCl_3$, 298 K), δ: 21.33 [$NCH_2C_6H_4(CH_3)$-3]; 48.32 [$NCH_2N(C{=}O)_2C_6H_4$]; 52.07 [$NCH_2C_6H_4(CH_3)$-3]; 113.51, 114.03, 124.36, 125.38, 127.46, 127.78, 128.98, 129.24, 130.08, 130.53, 131.07, 131.12, 132.13, 135.14 and 139.39 (Ar-*C*); 144.69 (N*C*HN); 166.81 [$NCH_2CH_2CH_2CH_2N(C{=}O)_2C_6H_4$]. HRMS $[L-Br]^+$ calcd for $C_{24}H_{20}N_3O_2$: 382.43, found m/z: 382.16. Elemental analysis for $C_{24}H_{20}N_3O_2Br$ (462.34 g/mol) (%): Found: C: 62.63; H: 4.52; N: 9.01. Anal. Calc. C: 62.35; H: 4.36; N: 9.09.

1,3-Bis(*N*-phthalimidomethyl)benzimidazolium bromide (3)

According to the same conditions and procedure as for **1**, **3** was synthesized from 1-(*N*-phthalimidomethyl)benzimidazole (1.29 g, 1 mmol) and *N*-(bromomethyl)phthalimide (1.12 g, 1 mmol) in DMF (4 mL).

Yield: 81%, mp: 324–325 ºC, color: white. IR: 1562.2 (CN); 1718.4 and 1782.1 (C=O); 2765.7 and 2867.9 (aliphatic, C-H); 3014.5 cm^{-1} (aromatic, C-H). ^{1}H NMR (400.13 MHz, $CDCl_3$, 298 K), δ: 6.42 [s, 2 H, $NCH_2N(C{=}O)_2C_6H_4$]; 7.75-8.18 (m, 12 H, Ar-*H*); 9.93 (s, 1 H, NC*H*N). $^{13}C\{^{1}H\}$ NMR (100 MHz, $CDCl_3$, 298 K), δ: 45.57 [$NCH_2N(C{=}O)_2C_6H_4$]; 110.68, 119.64, 123.60, 124.08, 124.35, 131.39, 132.40, 134.38, 134.85 and 141.29 (Ar-*C*); 143.55 (N*C*HN); 167.11 [$NCH_2CH_2CH_2CH_2N(C{=}O)_2C_6H_4$]. Elemental analysis for

$C_{25}H_{17}N_4O_4Br$ (517.33 g/mol) (%): Found: C: 57.75; H: 3.58; N: 10.74. Anal. Calc. C: 58.04; H: 3.31; N: 10.83.

1-(*N*-Phthalimidomethyl)-3-(2-morpholinoethyl)benzimidazolium bromide (4)

According to the same conditions and procedure as for **1**, **4** was synthesized from 1-(2-morpholinoethyl)benzimidazole (1.14 g, 1 mmol) and *N*-(bromomethyl)phthalimide (1.185 g, 1 mmol) in DMF (4 mL).

Yield: 62%, mp: 237–238 ºC, color: cream. IR: 1213.1 (C-O); 1554.5 (CN); 1726.2 and 1737.7 cm^{-1} (C=O). ^{1}H NMR (400.13 MHz, $CDCl_3$, 298 K), δ: 2.35 [t, *J*: 8.0 Hz, 4 H, $NCH_2CH_2N(CH_2CH_2)_2O$]; 3.16 [t, *J*: 8.0 Hz, 2 H, $NCH_2CH_2N(CH_2CH_2)_2O$]; 3.82 [t, *J*: 16.0 Hz, 4 H, $NCH_2CH_2N(CH_2CH_2)_2O$]; 4.97 (s, 2 H, $NCH_2CH_2N(CH_2CH_2)_2O$]; 6.58 [s, 2 H, $NCH_2N(CO)_2C_6H_4$]; 7.28-8.31 (m, 8 H, Ar-*H*); 10.83 (s, 1 H, NC*H*N). ^{13}C{^{1}H} NMR (100 MHz, $CDCl_3$, 298 K), δ: 43.76 [$NCH_2CH_2N(CH_2CH_2)_2O$]; 48.02 [$NCH_2CH_2N(CH_2CH_2)_2O$]; 53.16 [$NCH_2CH_2N(CH_2CH_2)_2O$]; 55.46 [$NCH_2CH_2N(CH_2CH_2)_2O$]; 66.19 [$NCH_2N(CO)_2C_6H_4$]; 113.31, 113.50, 124.33, 127.54, 127.76, 130.00, 131.15 and 135.21 (Ar-*C*); 145.26 (N*C*HN). HRMS $[L-Br]^+$ calcd for $C_{22}H_{23}N_4O_3$: 391.44, found m/z: 391.18. Elemental analysis for $C_{22}H_{23}N_4O_3Br$ (471.35 g/mol) (%): Found: C: 56.44; H: 5.02; N: 11.78. Anal. Calc. C: 56.06; H: 4.92; N: 11.89.

Dichloro[1-(*N*-phthalimidomethyl)-3-benzylbenzimidazol-2-ylidene](3-chloropyridine)palladium(II) (5)

Compound **5** was synthesized using 1-(*N*-phthalimidomethyl)-3-benzylbenzimidazolium bromide (0.2 g, 1 mmol), $PdCl_2$ (0.079 g, 1 mmol) and K_2CO_3 (0.31 g, 5 mmol) as a base in 3-chloropyridine (3 mL) at 80 °C for 16 h.

Yield: 60%, mp: > 350 ºC, color: yellow. IR: 1508.2 (CN); 1718.4 and 1733.9 (C=O); 2854.4, 2920.0, 2964.4 (aliphatic, C-H); 3014.5 cm^{-1} (aromatic, C-H). ^{1}H NMR (400.13 MHz, $CDCl_3$, 298 K), δ: 2.98 (s, 2 H, $NCH_2C_6H_5$); 6.17 [s, 2 H, $NCH_2N(CO)_2C_6H_4$]; 7.03-9.33 (m, 17 H, Ar-*H*). $^{13}C\{^{1}H\}$ NMR (100 MHz, $CDCl_3$, 298 K), δ: 49.26 [$NCH_2N(CO)_2C_6H_4$]; 54.20 ($NCH_2C_6H_5$); 111.47, 111.79, 111.89, 123.62, 123.84, 123.90, 124.91, 128.06, 128.27, 128.41, 128.88, 131.98, 132.49, 133.68, 134.42, 134.48, 135.75, 137.80, 150.64 and 151.72 (Ar-*C*; N*C*N); 167.65 and 167.74 [$NCH_2N(CO)_2C_6H_4$]. Elemental analysis for $C_{28}H_{21}N_4O_2Cl_3Pd$ (658.27 g/mol) (%): Found: C: 51.58; H: 3.46; N: 8.44. Anal. Calc. C: 51.09; H: 3.22; N: 8.51.

Dichloro[1-(*N*-phthalimidomethyl)-3-(3-methylbenzyl)benzimidazol-2-ylidene](3-chloropyridine)palladium(II) (6)

Complex **6** was synthesized from 1-(*N*-phthalimidomethyl)-3-(3-methylbenzyl)benzimidazolium bromide (0.2 g, 1 mmol), K_2CO_3 (0.299 g, 5 mmol) and $PdCl_2$ (0.077 g, 1 mmol) in 3-chloropyridine (3 mL).

Yield: 41%, mp: 242–243 ºC, color: cream. FT-IR: 1446.5 (CN); 1718.4 and 1722.3 (C=O); 3006.8 cm^{-1} (aromatic, C-H). ^{1}H NMR (400.13 MHz, $CDCl_3$, 298 K), δ: 2.33 [s, 3 H, $NCH_2C_6H_4(CH_3)$-3]; 6.12 [s, 2 H, $NCH_2C_6H_4(CH_3)$-3]; 6.73 [s, 2 H, $NCH_2N(C{=}O)_2C_6H_4$]; 7.11-9.15 (m, 16 H, Ar-*H*). $^{13}C\{^{1}H\}$ NMR (100 MHz, $CDCl_3$, 298 K), δ: 21.39 [$NCH_2C_6H_4(CH_3)$-3]; 49.06 [$NCH_2N(C{=}O)_2C_6H_4$]; 54.12 [$NCH_2C_6H_4(CH_3)$-3]; 111.47, 111.91, 123.58, 123.69, 123.91, 124.91, 125.10, 128.70, 129.05, 131.98, 132.50, 133.69, 133.76, 134.50, 135.52, 135.72, 137.78, 137.87, 138.70, 150.18, 150.64 and 151.72 (Ar-*C*; N*C*N); 167.66 and 167.75 [$NCH_2CH_2CH_2CH_2N(C{=}O)_2C_6H_4$]. HRMS $[M-Cl]^+$ calcd for

$C_{29}H_{23}N_4O_2Cl_2Pd$: 636.84, found m/z: 637.02. Elemental analysis for $C_{29}H_{23}N_4O_2Cl_3Pd$ (672.3 g/mol) (%): Found: C: 51.62; H: 3.71; N: 8.24. Anal. Calc. C: 51.81; H: 3.45; N: 8.33.

Dichloro[1,3-Bis(*N*-phthalimidomethyl)benzimidazol-2-ylidene](3-chloropyridine) palladium(II) (7)

Complex **7** was synthesized from 1,3-bis(*N*-phthalimidomethyl)benzimidazolium bromide (0.2 g, 1 mmol), $PdCl_2$ (0.069 g, 1 mmol) and K_2CO_3 (0.269 g, 5 mmol) as a base in 3-chloropyridine (3 mL) at 80 °C for 16 h.

Yield: 25%, mp: >350 ºC, color: yellow. IR: 1394.4 (CN); 1716.5 and 1778.2 (C=O); 2999.1 (aliphatic, C-H); 3051.2 and 3095.5 cm^{-1} (aromatic, C-H). ^1H NMR (400.13 MHz, $CDCl_3$, 298 K), δ: 6.61 [s, 4 H, $NCH_2N(C{=}O)_2C_6H_4$]; 7.28-9.33 (m, 16 H, Ar-*H*). $^{13}C\{^1H\}$ NMR (100 MHz, $CDCl_3$, 298 K), δ: 49.51 [$NCH_2N(C{=}O)_2C_6H_4$]; 112.73, 123.84, 123.95, 125.64, 126.59, 132.11, 134.54, 135.30, 148.70 and 150.80 (Ar-*C*; N*C*N); 167.91 [$NCH_2N(C{=}O)_2C_6H_4$]. HRMS $[M-Cl]^+$ calcd for $C_{30}H_{20}N_5O_4Cl_2Pd$: 691.84, found m/z: 691.99. Elemental analysis for $C_{30}H_{20}N_5O_4Cl_3Pd$ (727.29 g/mol) (%): Found: C: 49.71; H: 2.51; N: 9.57. Anal. Calc. C: 49.54; H: 2.77; N: 9.63.

Dichloro[1-(*N*-phthalimidomethyl)-3-(2-morpholinoethyl)benzimidazol-2-ylidene](3-chloropyridine)palladium (II) (8)

Complex **8** was synthesized from 1-(*N*-phthalimidomethyl)-3-(2-morpholinoethyl)benzimidazolium bromide (0.2 g, 1 mmol), K_2CO_3 (0.289 g, 5 mmol) and $PdCl_2$ (0.074 g, 1 mmol) in 3-chloropyridine (3 mL).

Yield: 33%, mp: 159–161 ºC, color: yellow. IR: 1261.4 (C-O); 1444.6 (CN); 1718.5 and 1722.3 (C=O); 2956.7 (aliphatic, C-H); 3058.9 cm^{-1} (aromatic, C-H). ^{1}H NMR (300.13 MHz, $CDCl_3$, 298 K), δ: 2.60 [t, *J*: 4.80 Hz, 4 H, $NCH_2CH_2N(CH_2CH_2)_2O$]; 3.13 [t, *J*: 6.90 Hz, 2 H, $NCH_2CH_2N(CH_2CH_2)_2O$]; 3.63 [t, *J*: 4.50 Hz, 4 H, $NCH_2CH_2N(CH_2CH_2)_2O$]; 4.88 (t, *J*: 7.50 Hz, 2 H, $NCH_2CH_2N(CH_2CH_2)_2O$]; 6.53 [s, 2 H, $NCH_2N(CO)_2C_6H_4$]; 7.19-9.07 (m, 12 H, Ar-*H*). $^{13}C\{^{1}H\}$ NMR (75 MHz, $CDCl_3$, 298 K), δ: 46.73 and 48.94 [$NCH_2CH_2N(CH_2CH_2)_2O$]; 53.95 [$NCH_2CH_2N(CH_2CH_2)_2O$]; 56.75 [$NCH_2CH_2N(CH_2CH_2)_2O$]; 61.50 [$NCH_2CH_2N(CH_2CH_2)_2O$]; 66.98 [$NCH_2N(CO)_2C_6H_4$]; 110.52, 111.94, 123.56, 123.85, 124.97, 131.99, 132.54, 134.28, 134.46, 135.29, 137.85, 150.60 and 151.71 (Ar-*C*; N*C*N); 165.62 and 167.71 [$NCH_2N(CO)_2C_6H_4$]. HRMS $[M-3Cl]^{+}$ calcd for $C_{27}H_{26}N_5O_3Pd$: 574.95, found m/z: 574.99. Elemental analysis for $C_{27}H_{26}N_5O_3Cl_3Pd$ (681.31 g/mol) (%): Found: C: 47.48; H: 3.61; N: 10.23. Anal. Calc. C: 47.60; H: 3.85; N: 10.28.

General procedure for the arylation reaction

All catalytic reactions were carried out under an air atmosphere. The purchased reagents were used without further purifications for C–C bond forming reactions. In a typical reaction, 2-*n*-butylfuran or 2-*n*-butylthiophene (2 mmol), aryl bromides (1 mmol), KOAc (1 mmol), PEPPSI Pd–NHC (1 mol %) and *N*,*N*-dimethylacetamide (DMAc, 2 mL) were added to a dry 25 mL Schlenk tube. The mixture was then stirred for different times at different temperatures. After the reaction was finished, the solvent in the medium was removed completely by vacuum and the remaining solid in the Schlenk tube was dissolved in hexane/diethyl ether (5:1), before it was purified over silica gel. The chemical characterisations of the products were made by GC or GC–MS. The yields were calculated according to aryl bromides as internal references. The results are given in Table 1.

General procedure for the Suzuki–Miyaura cross-coupling reaction

The preparation of biaryl products were performed in the presence of organoborane derivatives, aryl halides, synthesized compounds (**1**–**8**) and base in a DMF/H_2O mixture at different temperatures and times. When benzimidazolium salts were used, the reaction was performed using benzimidazolium salts, $Pd(OAc)_2$, aryl halides, boronic acid derivatives and base. When PEPPSI Pd–NHC complexes were used, the reaction was performed using Pd–NHC, aryl halides, boronic acid derivatives and base. In the standard work-up, a hexane/ethyl acetate (5:1) mixture was put into the reaction medium and was stirred for two minutes. The organic layer was separated and dried using anhydrous $MgSO_4$. The product was purified over silica gel by column chromatography using a short column and further analyzed using GC or GC–MS device. Yields were calculated according to aryl chlorides. The results are presented in Tables 2–4.
